# Association of serum levels of osteopontin and osteoprotegerin with adverse outcomes after endovascular revascularisation in peripheral artery disease

**DOI:** 10.1186/s12933-022-01605-6

**Published:** 2022-09-01

**Authors:** Nikolaos P. E. Kadoglou, Dimitrios Kapetanios, Emmanouil Korakas, Georgia Valsami, Nikolaos Tentolouris, Nikolaos Papanas, Vaia Lambadiari, Christos Karkos

**Affiliations:** 1 Medical School, University of Cyprus, 215/6 Old road Lefkosias-Lemesou, 2029 Aglatzia, Nicosia Cyprus; 2 Department of Vascular Surgery, University Hospital, LMU Munich, Munich, Germany; 3 2nd Department of Internal Medicine, Research Institute and Diabetes Centre, Athens University Medical School, Attikon University General Hospital, Athens, Greece; 4 School of Health Sciences, Department of Pharmacy, National & Kapodistrian University of Athens, Athens, Greece; 5 Diabetes Center, First Department of Propaedeutic Internal Medicine, Medical School, National and Kapodistrian University of Athens, Laiko General Hospital, Athens, Greece; 6 Diabetes Centre, Second Department of Internal Medicine, Democritus University of Thrace, 68100 Alexandroupolis, Greece; 7 5Th Department of Surgery, Aristotle University of Thessaloniki, Thessaloniki, Greece

**Keywords:** Osteoprotegerin, Osteopontin, Peripheral artery disease, MACE, Inflammation

## Abstract

**Background:**

Osteoprotegerin (OPG) and osteopontin (OPN) are vascular calcification inhibitors with a known role in the atherosclerotic and inflammatory process. We investigated their relationship with adverse outcomes (restenosis/adverse cardiovascular events) after endovascular revascularisation of patients with peripheral arterial disease (PAD).

**Methods:**

203 consecutive patients were enrolled in the PAD group (PADG) and 78 age and sex-matched subjects with less than two cardiovascular risk factors served as control group (COG). PADG underwent standard medical assessment at baseline and 12 months after the procedure. During follow up major adverse cardiovascular events (MACEs) including arterial restenosis with need for reintervention were documented and the PADG was divided accordingly into two subgroups.

**Results:**

During 12-month follow-up, 82 MACE were recorded (MACE subgroup). The rest of 124 PAD patients remained free of MACE (non-MACE subgroup). At baseline, OPG (9.89 ± 2.85 ng/ml vs 3.47 ± 1.95 ng/ml, p < 0.001) and OPN (79.99 ± 38.29 ng/ml vs 35.21 ± 14.84 ng/ml, p < 0.001) levels were significantly higher in PADG compared to COG, as well as in MACE subgroup compared to non-MACE subgroup (13.29 ± 3.23 ng/ml vs 10.86 ± 3 ng/ml and 96.45 ± 40.12 ng/ml vs 78.1 ± 38.29 ng/ml, respectively). An independent association of PAD with OPG and OPN was found in the whole patient cohort. Although OPG and OPN were significantly related to MACE incidence in the univariate analysis, multiple logistic regression analysis failed to detect any independent predictor of MACE within the PADG.

**Conclusion:**

Baseline high OPG and OPN levels were independently associated with PAD presence. Even higher levels of those biomarkers were detected among PAD patients with MACE, however, their prognostic role should be further clarified.

## Introduction

Peripheral artery disease (PAD) is one of the many complications of systemic atherosclerosis and it is accompanied by high rates of cardiovascular diseases (CVD) morbidity and mortality [1]. Endovascular revascularization has been considered the treatment of choice in most cases with excellent rates of long-term survival. However, vascular restenosis and post-interventional major adverse cardiovascular events (MACE) are still among the most frequent complications, especially in complex cases and high-risk populations, such as patients with diabetes or obesity [2]. As traditional indices such as the ankle-brachial index (ABI) have not been proven efficient to identify those patients at greatest risk for MACE, the search for other biomarkers involved in the inflammatory process has been under the spotlight in recent years [3].

Atherosclerosis is a complex procedure of different pathways. Among other cytokines, inflammation and calcification are known to be regulated also by well-known bone regulators, such as osteoprotegerin (OPG) and osteopontin (OPN) [4, 5]. OPG is a secretory basic glycoprotein and a member of the tumor necrosis factor (TNF) receptor superfamily, acting as an inhibitor of bone resorption [6]. It is produced by various organs and tissues (lungs, intestines, kidneys, bones, cardiovascular system) and by haematopoietic and immune cells. In the vasculature, as early animal studies showed, it inhibits vascular calcification [4, 7]. OPG may play a pivotal role in the development of vascular disease as in endothelial cells as it acts as an anti-apoptotic factor and it is expressed not only in non-diseased vessel wall, but also in early atherosclerotic lesions in human tissues [8]. Studies measuring serum OPG in PAD patients have shown conflicting results [9–13], On the other hand, the role of OPG as a risk factor for MACE has been more firmly established; however, the extent of this risk has not been consistent among all populations [14–16].

OPN is a secretory glycoprotein secreted by macrophages, osteoblasts, vascular smooth muscle cells (VSMC) and endothelial cells and is a regulator of bone metabolism and inflammation. It is involved in macrophage and VSMC migration and proliferation, promotion of vascular matrix metalloproteinase activation, cell-mediated immunity enhancement and inhibition of matrix calcium accumulation [5, 17]. The involvement of OPN in various steps of the inflammatory cascade and its strong relationship with endothelial function and integrity has revealed its role in the pathogenesis of the atherosclerotic process and, therefore, its association with states of chronic, low-grade inflammation, such as diabetes and CVDs [5, 18]. Although, a positive association between OPN and MACE has been indicated in many studies [5, 19–21], there is a paucity of data regarding the association between OPN and PAD [22–24]. The aim of the present study was to investigate the association of OPG and OPN levels with the presence of PAD, as well as with the incidence of long-term adverse outcomes (restenosis and/or cardiovascular events) in PAD patients undergoing lower limb endovascular revascularization.

## Methods

### Participants

In the present prospective, non-randomized study, we enrolled 203 consecutive patients with symptomatic, established PAD requiring endovascular revascularization (percutaneous transluminal angioplasty–PTA and/or stent placement) of any or both of their lower limbs (PAD group–PADG). PAD diagnosis was based on ankle-brachial index (ABI < 0.80), high degree stenosis (> 70%) of peripheral arteries documented by duplex ultrasound and symptoms of intermittent claudication, rest pain and/or skin lesions according to the TASC guidelines [25]. Thereafter, angiographically-demonstrated stenosis (≥ 70% of the cross-sectional area) of the iliac arteries or below was demonstrated.

We excluded patients with any of the following concurrent conditions/diseases interfering with the expression of vascular calcification or inflammatory mediators, like active infection, wet gangrene, severe kidney or liver impairment, cancer, osteoporosis, cardiovascular events within the recent month, atrial fibrillation, any major surgery or severe trauma (e.g. fracture without surgery) over the last 3 months and chronic inflammatory or autoimmune diseases. Patients were also excluded if they were planned for open surgery or hybrid (open surgery combined with endovascular revascularization) due to anatomical reasons. The study was approved by the local Research and Ethics committee. A written informed consent was obtained prior to patients’ participation.

We also recruited 78 age- and sex-matched subjects with less than 2 classical cardiovascular risk factors (hypertension, diabetes, hyperlipidemia, smoking, family history of premature CAD) who served as control group (COG). The latter participants were selected among visitors of our outpatient clinics for their routine check-up. As a prerequisite, those individuals were free from any chronic CVD (coronary artery disease–CAD or PAD) based on test of myocardial ischemia and ultrasound of peripheral arteries within the last 2 years.

### Study design

PADG underwent standard medical assessment at baseline and 12 months after intervention, while COG only at baseline. The standard assessment included clinical examination, blood sampling and ultrasound examination of both lower-limb arteries. Prior to intervention, lipid-lowering and anti-platelet therapy (LDL-C target < 100 mg/dl) were prescribed to all patients, unless it was contra-indicated. Following endovascular revascularization procedure, with provisional stent employment, all patients remained on dual antiplatelet therapy (clopidogrel 75 mg plus acetyl salicylic acid 100 mg) for 6 months and then on life-long clopidogrel therapy, unless it was deemed medically necessary to select another anti-platelet agent. Physicians were asked to optimize the pharmaceutical therapy of all PAD patients and to encourage them to incorporate a healthier life-style. During the 12-month follow-up, we recorded the occurrence of MACE like acute coronary events, coronary revascularization and peripheral artery restenosis requiring re-revascularization (either endovascular or open surgery) based on medical records. Re-revascularization was conducted in symptomatic patients with angiographically stenosis ≥ 50% found either in stents or at lesion sites of previously balloon angioplasty. All cases were peer-reviewed by a multi-disciplinary team (vascular surgeons and cardiologists) and the decision of fulfilling MACE criteria was taken based on medical records. Patients with at least one MACE were assigned to the MACE subgroup and the rest to the non-MACE subgroup for analysis purposes.

### Clinical examination

Using a structured questionnaire, we obtained medical information about medications and co-morbidities. During clinical examination, we measured blood pressure (BP), weight and height to calculate body-mass index (BMI). BP was measured twice, after keeping participants at a sitting position for 15 min. There was a 5-min interval between the two measurements and the mean value was estimated. Patients with BP > 140/90 mmHg or on anti-hypertensive medications were considered hypertensive. Patients with fasting plasma glucose ≥ 126 mg/dl or HbA1c > 6% or already on anti-diabetic medications were considered as diabetic. Similarly, hyperlipemia was defined as the existence of LDL-C > 160 mmHg or current therapy with lipid-lowering drugs.

### Blood assays

After overnight fasting, blood samples were obtained and glycemic and lipid parameters were immediately assayed in an automatic enzymatic analyzer (Olympus AU560, Hamburg, Germany). The glycated haemoglobin (HbA1c) was determined by high-performance liquid chromatography (Menarini Diagnostics, Florence, Italy) only in diabetic patients. A blood quantity was collected and, after a centrifugation at 2500 rpm for 10 min, frozen serum samples were stored (− 80 °C) until analysis in the same assay. Serum OPN and OPG were assayed using quantikine immunoassay EIA kits (R&D Systems Inc., Minneapolis, USA and Metra, San Diego, USA). The intra- and inter-assay coefficients of variance were 2.6% and 5.7% for OPN and 7% and 6.8% for OPG, respectively. The high-sensitivity C-Reactive Protein (hsCRP) levels were measured with latex-enhanced immunonephelometry (Dade Behring, Marburg, Germany).

### Statistical analysis

Normality of distribution was assessed with Kolmogorov–Smirnov test. Results of normally distributed continuous variables were expressed as the mean value ± SD. Log transformation was used in case of non-normal distributed variables. Continuous and categorical variables were compared using the student’s t-test and chi-square test, respectively. Pearson’s correlation coefficient was calculated to determine the strength of the association of PAD presence or MACE with continuous variables. After univariate analysis, variables showing a significant correlation with PAD or MACE entered a logistic multiple regression analysis to check for independent associations. A two-tailed p value < 0.05 was considered as statistically significant. The computer software package SPSS (version 25.0; SPSS Inc, Chicago, IL, USA) was used for statistical analysis.

## Results

### Baseline comparisons

At baseline, significant differences between PADG and COG were observed in systolic BP, smoking rate, HDL, kidney function indices (creatinine, glomerular filtration rate–GFR), hsCRP, OPG and OPN levels (p < 0.05) (Table 1). Despite the high prevalence of diabetes and hyperlipidemia in PAD patients, fasting blood glucose and most lipid parameters, respectively, did not significantly differ from controls due to optimal therapy. Notably, 10 statin-free PAD patients had significantly higher levels of both OPN and OPG levels than their statin-treated counterparts or controls (data not shown).

**Table 1:** Clinical and laboratory characteristics of both, peripheral artery disease group (PADG) and control group (COG) at baseline

	PADG (n = 203)	COG (n = 78)	*P value*
Age (y)	77 ± 15	71 ± 13	0.0256
Males, n (%)	174 (85.7)	65 (83.3)	0.949
Smoking, n (%)	50 (24.6)	14 (18.2)	0.002
Hypertension, n (%)	151 (74.4)	13 (16.7)	< 0.001
Dyslipidemia, n (%)	197 (97)	36 (46.2)	< 0.001
Statins, n (%)	193 (95.1)	20 (25.6)	< 0.001
ABI	0.51 (0.11)	1.1 (0.1)	< 0.001
Diabetes, n (%)	81 (39.9)	7 (9)	< 0.001
BMI (kg/m^2^)	28.12 ± 3.67	26.5 ± 3.98	0.101
SBP (mmHg)	138 ± 22	121 ± 13	< 0.001
DBP (mmHg)	85 ± 10	77 ± 8	0.387
TChol (mg/dl)	175 ± 53	158 ± 49	0.076
HDL-C (mg/dl)	40 ± 9	49 ± 12	< 0.001
LDL-C (mg/dl)	112 ± 43	89 ± 22	0.065
TG (mg/dl)	114 ± 58	101 ± 33	0.405
Creatinine (mg/dl)	1.31 ± 0.41	0.98 ± 0.22	0.021
GFR (mL/min/1.73 m^2^)	51 ± 15	98 ± 9	0.002
FPG (mg/dl)	139 ± 41	100 ± 15	< 0.001
HbA1c (%)^a^	7.5 ± 1.3	6.5 ± 0.8	-
hsCRP (mg/L)	7.88 ± 2.11	1.06 ± 0.39	< 0.001
OPN (ng/ml)	79.99 ± 38.29	35.21 ± 14.84	< 0.001
OPG (pmol/L)	9.89 ± 2.85	3.47 ± 1.95	< 0.001

Data are expressed as means ± SD

*n* number of patients; *BMI* body-mass index; *SBP* systolic blood pressure, *DBP* diastolic blood pressure; *TChol* total cholesterol; *TG* triglycerides; *FPG* fasting plasma glucose; *hsCRP* high-sensitivity C-Reactive Protein; *OPN* osteopontin; *OPG* osteoprotegerin

^a^HbA1c was measured only in diabetic patients. due to the very small subgroup of diabetic controls we did not statistical difference for HbA1c

*P value* of differences between groups

At baseline, MACE subgroup appeared with significantly higher smoking rate, systolic blood pressure, hsCRP and LDL-C, OPN, OPG levels than non-MACE subgroup. No other significant differences were detected between those subgroups at baseline in the rest of variables, (p > 0.05) (Table 2).

**Table 2:** Clinical and laboratory data at baseline and at the end of the study of MACE and non-MACE subgroups (MACE- major adverse cardiovascular events)

	Groups				P value
	MACE (N = 75)		Non-MACE (N = 124)		
	Baseline	12 months	Baseline	12 months	
Age (y)	81 ± 15	–	76 ± 12	–	0.432
Males, n (%)	60 (80)		101 (81.4)		0.951
Smoking, n (%)	32 (42.7)	18 (24)	18 (14.5)#	9 (7.5)	< 0.001
Hypertension, n (%)	58 (77.3)	–	90 (72.6)	–	0.383
Dyslipidemia, n (%)	73 (97.3)	–	119 (96)	–	0.909
Statins, n (%)	73 (97.3)	74 (98.7)	118 (95.2)	121 (97.6)	0.943
ABI	0.49 (0.09)	–	0.54 (0.09)	–	0.799
Diabetes, n (%)	34 (45.3)	–	45 (36.3)	–	0.206
BMI (kg/m^2^)	27.98 ± 3.72	27.56 ± 3.55	28.21 ± 3.9	28 ± 3.7	0.922
SBP (mmHg)	144 ± 24	140 ± 25	135 ± 24#	134 ± 28	0.108
DBP (mmHg)	85 ± 8	84 ± 7	85 ± 9	85 ± 10	0.998
TChol (mg/dl)	176 ± 49	167 ± 46	165 ± 41	159 ± 46	0.967
HDL-C (mg/dl)	38 ± 8	39 ± 7	41 ± 11	42 ± 9	0.781
LDL-C (mg/dl)	130 ± 43	104 ± 37*	102 ± 35#	92 ± 22*	0.043
TG (mg/dl)	132 ± 61	120 ± 61	109 ± 42	101 ± 42	0.070
Creatinine (mg/dl)	1.42 ± 0.55	1.35 ± 0.52	1.27 ± 0.47	1.20 ± 0.37	0.980
GFR (mL/min/1.73 m^2^)	47 ± 12	50 ± 12	55 ± 19	58 ± 19	0.926
FPG (mg/dl)	155 ± 45	135 ± 45*	146 ± 41	128 ± 28	0.900
HbA1c (%)^a^	7.9 ± 1.4	7.3 ± 1.1	7.3 ± 1.3	6.9 ± 0.9	0.131
hsCRP (mg/L)	8.05 ± 2.67	4.79 ± 1.27	5.55 ± 2.12#	2.98 ± 1.05	< 0.001
OPN (ng/ml)	96.45 ± 40.12	78.1 ± 38.29	73.05 ± 29.88#	51.66 ± 18.77*	< 0.001
OPG (pmol/L)	13.29 ± 3.23	10.86 ± 3*	9.04 ± 3.56#	6.12 ± 2.34*	< 0.001

Data are expressed as means ± SD

*n* number of patients; *BMI* body-mass index; *SBP* systolic blood pressure *DBP* diastolic blood pressure; *TChol* total cholesterol; *TG* triglycerides; *FPG* fasting plasma glucose; *hsCRP* high-sensitivity C-Reactive Protein; *OPN* osteopontin; *OPG* osteoprotegerin

P value of changes of variables between groups

*p < 0.05 of changes of variables within groups

^#^p < 0.05 of differences of variables between groups at baseline

^a^HbA1c was measured only in diabetic patients

#### Follow-up results

Eight patients were lost during follow-up for personal reasons or were unable to return for follow-up assessment. During the 12-month follow-up period, we recorded 82 MACE in 75 PAD patients (MACE subgroup): 6 patients experienced an acute cardiovascular event and another 69 underwent at least one new endovascular intervention or open surgery revascularization due to restenosis. Four patients with MACE eventually died due to complications. The rest of 124 PAD patients remained free of MACE for the same time period (non-MACE subgroup). Therefore, 191 PAD patients completed the study and their clinical and laboratory data at baseline and at the end were included in the analysis (Table 2).

#### Correlations

**Fig. 1:**
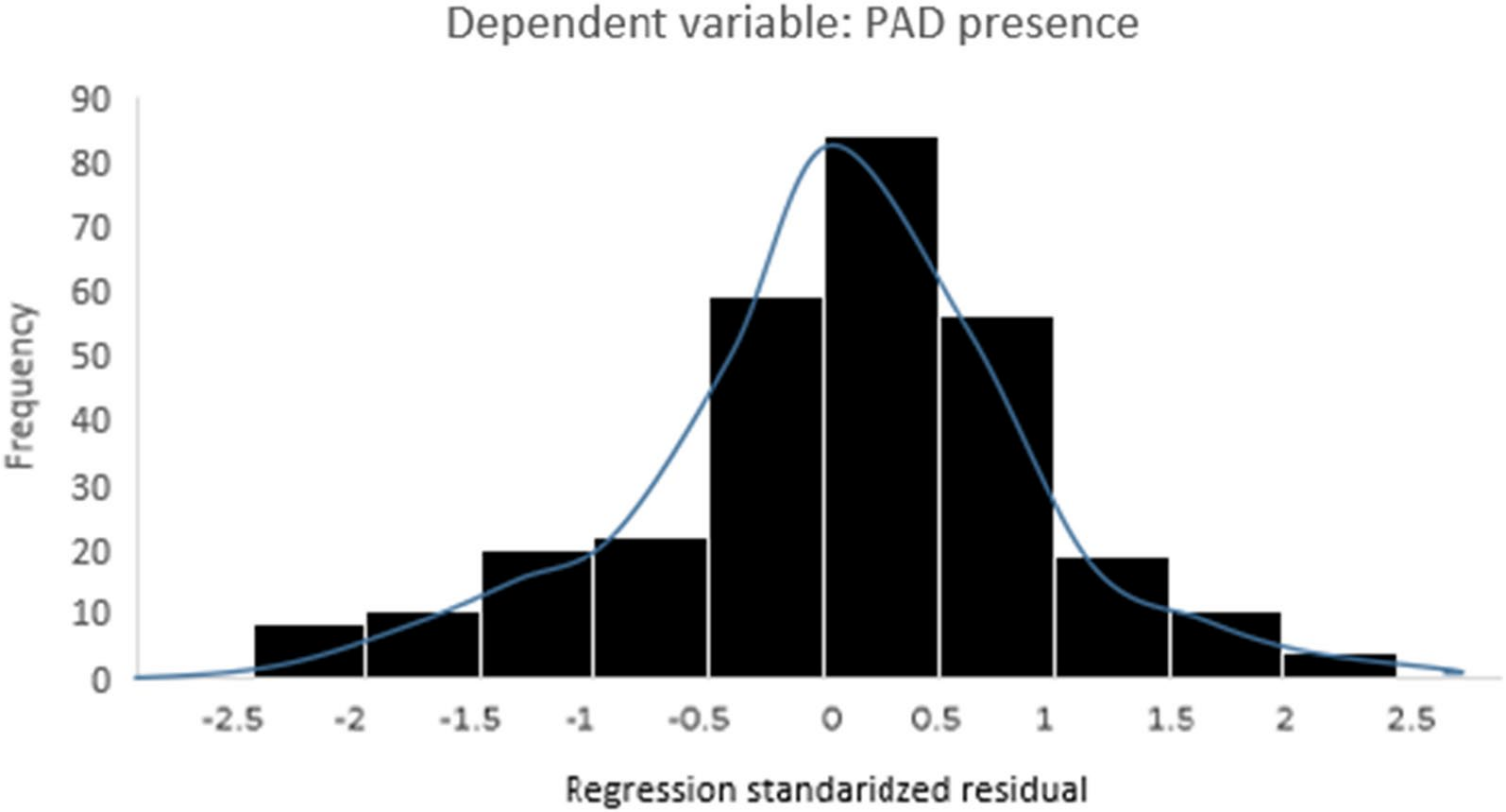
Histogram of regression standardized residuals for PAD presence within the whole study cohort (PAD patients and controls)

In the whole study cohort, the presence of symptomatic PAD was significantly associated with age, hsCRP, OPN and OPG levels in univariate analysis (p < 0.05). Entering a multiple logistic regression analysis adjusted for age, an independent association of PAD presence with hsCRP, OPN, OPG and smoking was found (R^2^ = 0.310, p = 0.013) (Table 3 and Fig. 1).

**Table 3:** Standard multiple regression analysis of PAD presence (dependent variable) and other independent variables within the whole study cohort (PAD patients and controls)

	PAD		
	β	95% CI	P value
OPN	0.225	0.112–0.385	0.035
OPG	0.297	0.202–0.411	< 0.001
hsCRP	0.176	0.023–0.412	0.043
Smoking	0.331	0.090–0.775	0.006

*CI* confidence interval; *OPN* osteopontin; *OPG* osteoprotegerin; *hsCRP* high-sensitivity C-Reactive Protein

Following similar methodology, we searched for the independent predictors of MACE within PADG. In univariate analysis, OPN, OPG, hsCRP and smoking were significantly related to MACE incidence (p < 0.05). However, multiple logistic regression analysis failed to detect any independent predictor of MACE within PADG.

## Discussion

In our study, patients with significant lower-limb PAD requiring endovascular revascularization appeared with significantly higher circulating levels of hsCRP, OPN and OPG compared to age- and sex-matched individuals without CVD. Multivariate analysis confirmed an independent association of those biomarkers with the presence of PAD. Among PAD patients, those developing MACE during the 12-months follow-up, had even higher hsCRP, OPN and OPG circulating levels, than non-MACE group, but we failed to detect their prognostic value in regression analysis.

### Osteoprotegerin and atherosclerotic diseases

Regarding OPG, our results are in line with most previous studies. Among 98 patients with diabetes with or without PAD, OPG was independently associated with > twofold risk of PAD (odds ratio- OR:2.26) [13]. Within type 2 diabetes mellitus (T2DM) population, higher OPG levels have been found in PADG compared to COG (p < 0.001) [26]. Notably, O’ Sullivan et al. [9] confirmed higher serum OPG in PAD patients than controls, regardless of the co-existence of T2DM. The association between high OPG and PAD seems to be also independent of race, in a large-scale study including 1291 African–Americans and 1152 non-Hispanic whites, while a strong and consistent positive association has also been reported in patients in peritoneal dialysis [27]. On the other hand, a small number of studies has failed to demonstrate such association. In details, a study enrolling T2DM patients without known CVD, demonstrated significantly increased OPG levels in patients with carotid and peripheral arterial disease compared to controls, but this association remained significant only for carotid disease after adjustment for age, glycosylated hemoglobin (HbA1c) and urine albumin-to-creatinine ratio [8]. Ziegler et al. [10], found no difference in OPG concentrations between 67 patients undergoing PTA of PAD and 94 age-matched healthy controls. However, in order to properly interpret these seemingly contradicting results, it must be noted several drawbacks in those studies, like the absence of data about ABI measurement, glycaemic status or CVD history which questioned the severity of PAD and blurred its association with OPG.

In our study, although elevated baseline OPG levels were associated with the incidence of cardiovascular complications or restenosis requiring re-revascularisation among PAD patients; this association was not confirmed in multiple regression analysis. Previous research data favour a causal relationship between OPG and MACE, but the evidence is not robust. Similarly, Biscetti et al. [14] showed the association between baseline levels of various inflammatory cytokines (OPG among them) and failure of lower extremity endovascular revascularisation in patients with diabetes, PAD and chronic limb-threatening ischemia (CLTI). A significant linear association between the lowest and highest quartiles of OPG and the risk of major adverse limb events (MALE: acute limb ischemia, major vascular amputations and urgent limb revascularisation) as well as the risk of MACE was found even after adjusting for other risk factors in multivariate models. The prognostic value of OPG has been implicated in patients with stable angina pectoris, but independent effects were limited to levels above the 90th percentile [28], while higher OPG levels have also been associated with higher incidence of internal carotid stenosis in diabetics compared to those without stenosis [29]. In a recent meta-analysis of 9 studies including 26,442 participants of general population, those in the top third of OPG concentration had higher combined risk ratio for CVD (1.83), CAD (1.72), and stroke (1.58), compared to individuals at the bottom third of OPG concentration, implicating its predictive power [16]. However, when the meta-analysis focused only on high-risk populations, namely diabetes mellitus, chronic kidney disease (CKD), pre-existing heart disease or recent acute coronary syndromes, this predictive power was significantly attenuated [15]. Therefore, those inconsistent results could be explained by the heterogeneity of concomitant treatment regimens; for instance, some antidiabetic agents and statins are well-known to affect circulating OPG levels [30, 31]. Notably, within our PADG, a small subgroup of 10 statin-free patients had higher OPG levels compared to statin-treated PAD patients. In addition, OPG levels vary among different diseases, such as PAD, diabetes and CKD. We cannot exclude that its association with MACE can be attenuated also because of the dominance of other risk factors more relevant to CVD risk, such as metabolic syndrome, smoking or family history. In the majority of previous studies, bone status was not assessed, which could have seriously affected OPG levels. Hence, the prognostic value of OPG remains under investigation.

### Osteoprotegerin and atherosclerotic pathophysiological mechanisms

Whether OPG mediates or protects atherosclerosis is unclear. On the one hand, OPG acts as a soluble decoy receptor for the receptor activator of nuclear factor-B ligand (RANK-L) and the TNF-related apoptosis inducing ligand (TRAIL) [32]. It has been postulated a compensatory, yet often failed, self-defence response of OPG to atherogenesis. On the other hand, OPG can exert pro-inflammatory effects; by increasing macrophage infiltration and leukocyte adhesion to endothelial cells, and sensitizing them to the effects of TNF-alpha via upregulation of angiopoietin-2 [33, 34]. RANKL promotes osteogenic differentiation of vascular smooth muscle cell and stimulates the release of various pro-inflammatory cytokines, such as matrix metalloproteinase (MMP)-9, while TRAIL is a potent activator of apoptosis. Therefore, the action of OPG exerts an anti-inflammatory, anti-apoptotic and anti-calcifying effect [6, 35]. In addition, by blocking RANK-L, OPG reduces nitric oxide synthase production and leads to decreased vascular dilatation and endothelial dysfunction [36]. Finally, a role in plaque destabilization has been implied, however, the study data remain rather conflicting [6].

### Osteopontin and atherosclerotic diseases

Regarding the relationship between OPN and PAD, literature data are scarce, but they generally favour a positive association. Recently, a cross-sectional study enrolling 70 individuals with T2DM and 66 controls, showed significantly higher plasma OPN levels in PAD patients compared to PAD-free counterparts (p < 0.001), regardless of their glycaemic status [23]. In addition, OPN was negatively associated with ABI (Spearman’s r =  − 0.245 in the whole sample), indicating a positive association with PAD severity. It must be noted, that a range of OPN levels that could be considered normal has not yet been determined, with median values reported among healthy individuals ranging between 20.25 and 55.1 ng/mL.

A significant amount of evidence exists regarding the association of OPN with MACE, although these data derived mainly from CAD or stroke populations. In a sub-analysis of the PEACE trial, where 3567 patients with stable CAD were studied, OPN was significantly associated with the composite primary endpoint of cardiovascular death, non-fatal myocardial infarction and hospitalization for heart failure, even after adjustment for relevant co-variates [37]. However, a secondary analysis showed that this association was predominantly driven by the hospitalization for heart failure. In PAD patients, Lin et al. [22] reported high OPN levels as strong predictors of all-cause death, with the optimal cutoff concentration for predicting mortality being 126 ng/ml [22]. In agreement, we observed a positive association between OPN and PAD, which was lost after multiple logistic regression analysis. An even stronger association between OPN and MACE was revealed in a similar population by Georgiadou et al. (HR: 2.88) for OPN levels > 55 ng/ml [19], but those levels were considerably lower than the median values in our cohort and the study by Lin et al. [22]. OPN has also been associated with restenosis after coronary revascularization [38], but this has not been a consistent finding [39]. In 80 patients under chronic hemodialysis, OPN levels were associated with the severity of carotid stenosis [40], and in a large cohort of 3545 ischemic stroke patients, elevated OPN at baseline was a risk factor for adverse clinical outcomes at 1 year after ischemic stroke, rendering OPN a possible prognostic biomarker [41]. Overall, the existing data, predominantly from CAD studies and to a lesser extent from PAD trials, are controversial concerning the prognostic value of OPN. Methodological factors, such as sample size and follow-up duration, or the concomitant use of lipid-lowering or antidiabetic agents are possible explanations for those inconsistent results.

### Osteopontin and atherosclerotic pathophysiological mechanisms

OPN mediates its vascular effects through complex pathways. Although hardly detectable under physiological conditions, OPN expression is often increased 20–50-fold in response to acute ischemic events, being a key player in the immune and inflammatory response [5]. More specifically, OPN promotes chemotaxis and recruitment of macrophages, as well as smooth muscle cell proliferation, thus contributing to increased inflammation and post-ischemic neovascularization. States of chronic, low-grade inflammation and endothelial dysfunction, such as hyperglycemia, hypoxia or dyslipidemia, have also been shown to increase OPN expression in VSMCs, which in turn exacerbates oxidative stress and inflammation, leading to a vicious cycle. Apart from inflammation; increased levels of OPN have been found within human atherosclerotic plaques from the aorta, carotid and coronary arteries [42]. In mouse models, OPN overexpression significantly increased atherosclerotic lesions [43], while depletion of one or both OPN genes attenuated angiotensin II–accelerated atherosclerosis [44] and treatment with liraglutide significantly attenuated OPN expression in a rat model of metabolic syndrome [45]. On the other hand, OPN is a potent inhibitor of vascular calcification, an effect which may be considered vasculoprotective. However, the anti-calcifying effect of OPN is not consistent, but it actually relies heavily on its phosphorylation status [46].

### Osteoprotegerin and osteopontin and chronic kidney disease

In our study, we excluded patients with severe CKD, however 23% of our PAD patients appeared with mild or moderate (stages 2–4) CKD. Regarding the increase of OPG and OPN levels along renal function worsening [47], we cannot rule out the contribution of renal impairment to the higher serum levels of those biomarkers in PADG than COG. On the other hand, a significant difference in OPG and OPN serum levels persisted between MACE and non-MACE groups, despite the absence of significant difference in kidney function. Moreover, univariate and multivariate analysis did not show any significant correlation of creatinine and GFR with OPG and OPN. Hence, the interplay of CKD with OPG, OPN is not clear in our PAD cohort and its impact on prognosis seems undetermined.

### Limitations

The relatively small sample size and the short follow-up duration are among the most common limitations of our study. Furthermore, no assessment of bone status was conducted. Another important drawback is the fact that there is a wide variety of factors affecting circulating levels of biomarkers, which prevented the demonstration of a cause-effect relationship between examined biomarkers and clinical outcomes. Moreover, the vast majority of our patients was already on optimal pharmaceutical therapy, which might have influenced the levels of used biomarkers and clinical outcomes, undermining the predictive power of biomarkers. Another limitation was the relatively high smoking rate among PAD patients, driving the restenosis rate. However, this is common in clinical practice and reflects an unavoidable situation.

## Conclusions

Serum levels of OPG and OPN were elevated in patients with PAD undergoing revascularization compared to control subjects. Furthermore, we found an independent association of OPG, OPN, hsCRP and smoking with PAD presence. Most importantly, we observed higher levels of both OPN and OPG at baseline before PTA in PAD patients who experienced MACE during follow-up. Although the exact pathophysiological contribution of OPG and OPN to peripheral atherosclerotic disease is yet to be elucidated, our findings suggest that OPG and OPN could serve as possible biomarkers of PAD progression/prognosis after endovascular revascularization. Further studies are warranted to enlighten the role of OPG and OPN as well as their true predictive role in cardiovascular events in PAD.

## Data Availability

The datasets used and/or analyzed during the current study are available from the corresponding author on reasonable request.

## Abbreviations

ABI: Ankle-brachial index
BMI: Body-mass index
BP: Blood pressure
CAD: Coronary artery disease
CLTI: Chronic limb-threatening ischemia
CVD: Cardiovascular disease
DBP: Diastolic blood pressure
FPG: Fasting plasma glucose
HbA1c: Glycated hemoglobin
HDL: High-density lipoprotein
HR: Hazard ratio
hsCRP: High-sensitivity C-Reactive Protein
LDL-C: Low-density lipoprotein cholesterol
MACE: Major adverse cardiovascular event
MALE: Major adverse limb events
MMP: Matrix metalloproteinase
OPG: Osteoprotegerin
OPN: Osteopontin
PAD: Peripheral artery disease
PTA: Percutaneous transluminal angioplasty
RANKL: Nuclear factor-B ligand
SBP: Systolic blood pressure
T2DM: Type 2 diabetes mellitus
TChol: Total cholesterol
TG: Triglycerides
TNF: Tumor necrosis factor
TRAIL: TNF-related apoptosis inducing ligand
VSMC: Vascular smooth muscle cell
